# Big Data Are Only as Good as the People and the Processes That Create Them: The EUROlinkCAT Success Story

**DOI:** 10.1111/ppe.70014

**Published:** 2025-03-12

**Authors:** Babak Khoshnood

**Affiliations:** ^1^ INSERM U1153, Obstetric, Perinatal, Paediatric Life Course Epidemiology ‐ Center for Research on Epidemiology and Statistics Sorbonne Paris Cité, Inserm Université Paris Cité Paris France

1

It would be short‐sighted and archaic to deny that, with the increasing availability of big data and advancement in artificial intelligence, particularly machine learning and large language models, we have entered a new era of public health, medical research and scientific discovery. However, the current enthusiasm and, at times, even a certain degree of hype for these developments can also result in unintended negative consequences. This can happen if we are not careful during the planning and execution of big data construction, mainly when using and analysing administrative databases, whether by humans or machines.

The paper by Loane et al. [1] about the lessons learned from the EUROlinkCAT study is an excellent example of putting such concerns in a positive perspective. It underscores the added value of undertaking an ambitious linkage study that spans several different data sources in multiple countries. It also illustrates that one cannot underestimate the knowledge, experience, training and efforts required for creating and analysing high‐quality, standardised linked big data that allow addressing multiple questions and objectives. The paper is a good example of what the economists call a ‘positive externality’ of the project, as the experiences of those who led the project and subsequently analysed the data have resulted in recommendations that benefit us all.

EUROlinkCAT was funded by the European Union's Horizon 2020 Research and Innovation programme to assess mortality and morbidity outcomes in children with congenital anomalies. It was a population‐based cohort study of almost 100,000 children born between 1995 and 2014 with congenital anomalies. Seventeen population‐based European registries who are members of the European Surveillance of Congenital Anomalies (EUROCAT with the Central Registry at the Joint Research Centre in Italy, https://eu‐rd‐platform.jrc.ec.europa.eu/eurocat_en), including Belgium, Croatia, Denmark, Finland, France, Germany, Italy, Malta, Netherlands, Norway, Portugal, Spain, Ukraine and the United Kingdom, participated in the EUROlinkCAT study.

The EUROCAT registries use multiple data sources for case ascertainment and verification of the diagnoses with codes and data standardised according to EUROCAT guidelines. For the EUROlinkCAT study, routinely available data, including birth and death records, prescription and hospital discharge data, were harmonised to common data models. Central analysis scripts then produced aggregate tables for the analyses. Not all registries could provide the entire dataset, including hospital discharge and prescription data. Nevertheless, all 17 registries linked their standardised congenital anomalies data to national or regional mortality records. To date, 35 papers have been published on the basis of this data, which have partly informed the recommendations issued in the article by Loane et al.

The most important recommendation of Loane et al. [1] concerns terminations of pregnancy for foetal anomaly (TOPFA). These currently account for approximately 20% of all cases, and in certain registries, including ours in Paris, TOPFA accounts for almost one‐third of all cases [2]. Hence, surveillance of the prevalence and assessments of the outcomes of congenital anomalies are incomplete unless TOPFA are included [3]. Loane et al. recommend that all TOPFA cases be recorded in the mother's healthcare records. Moreover, they note that the current WHO ICD‐10 system is inadequate for recording TOPFA following prenatal diagnosis of a congenital anomaly, and they suggest ways of addressing this problem.

Another critical issue is that many national data systems currently lack a permanent, unique identification number assigned at birth that would enable accurate linkage across multiple healthcare and other databases. Inaccurate and heterogeneous coding can be a significant challenge and barrier to using administrative databases to address questions related to congenital anomalies. This is particularly problematic in cases such as congenital heart defects [4, 5], which involve complex diagnoses and numerous codes. For example, the preferred coding system for congenital heart defects among paediatric cardiologists is the extended version of the International Pediatric Congenital Cardiac Code, which includes over 10,000 individual codes [6]. Another critical example is hypospadias, where coding has long been recognised as a major issue for effective surveillance of this anomaly [7]. To address these issues or to mitigate their impact, the authors recommend using extended versions of the ICD‐10 for coding rare anomalies or adapting other coding systems better suited for characterising particularly complex anomalies.

Finally, national and European policies that prevent the release of results based on a small number of children due to confidentiality concerns severely hinder the visibility of rare anomalies, the study of their causes, and their impact on individual patients and public health. The authors are correct in advocating for adopting policies that allow the release of small numbers to named, trusted researchers with disclosure agreements, thereby enabling more effective surveillance and evaluation studies on rare anomalies, especially in smaller registries or countries.

Administrative and other routinely available healthcare data certainly offer advantages in terms of time and costs. For example, the Système National des Données de Santé (SNDS) in France has provided valuable data that have been published in a variety of subjects [8, 9, 10]. These include post‐marketing surveillance studies of medication use and adverse effects, healthcare costs and clinical care pathways.

Nonetheless, the EUROlinkCAT study and the paper by Loane et al. show that significant barriers remain in using administrative databases for surveillance or research purposes in congenital anomalies. Although improvements are possible and will come over time, there is a need for continued funding and operation of population‐based registries of congenital anomalies that can provide consistent, high‐quality, standardised data over the long term. This is the only feasible means to safeguard effective surveillance of congenital anomalies, assess teratogens, including medications and environmental factors, and evaluate preventive health policies and the impact of changes in medical and surgical care in this field. Otherwise, it would be unrealistic to think that stakeholders and policymakers will have access to the necessary information for devising and evaluating health policies related to congenital anomalies, many of which are classified as Rare Diseases by the European Union.

In these disconcerting times of rather indiscriminate public spending cuts, it is essential to remember that you get what you pay for—and indeed, there is no free lunch.

## Author Contributions

The author takes full responsibility for this article.

## Disclosure

The author has nothing to report.

## Conflicts of Interest

The author declares no conflicts of interest.

## Data Availability

The author has nothing to report.
